# The C-terminal domain of TPX2 is made of alpha-helical tandem repeats

**DOI:** 10.1186/s12900-016-0070-8

**Published:** 2016-10-26

**Authors:** Luis Sanchez-Pulido, Laurent Perez, Steffen Kuhn, Isabelle Vernos, Miguel A. Andrade-Navarro

**Affiliations:** 1 MRC Human Genetics Unit, Institute of Genetics and Molecular Medicine, The University of Edinburgh, Western General Hospital, Crewe Road, Edinburgh, EH4 2XU UK; 2 Center for Genomic Regulation, C/ Dr Aiguader 88, 08003 Barcelona, Spain; 3 Institute for Research in Biomedicine, Università della Svizzera italiana, Via Vincenzo Vela 6, CH-6500 Bellinzona, Switzerland; 4 Faculty of Biology, Johannes-Gutenberg University, Gresemundweg 2, 55128 Mainz, Germany; 5 Institute of Molecular Biology, Ackermannweg 4, 55128 Mainz, Germany

**Keywords:** TPX2, Protein sequence tandem repeats, Protein sequence analysis, Protein structure prediction, Alpha-solenoid, Circular Dichroism

## Abstract

**Background:**

TPX2 (Targeting Protein for Xklp2) is essential for spindle assembly, activation of the mitotic kinase Aurora A and for triggering microtubule nucleation. Homologs of TPX2 in Chordata and plants were previously identified. Currently, proteins of the TPX2 family have little structural information and only small parts are covered by defined protein domains.

**Methods:**

We have used computational sequence analyses and structural predictions of proteins of the TPX2 family, supported with Circular Dichroism (CD) measurements.

**Results:**

Here, we report our finding that the C-terminal domain of TPX2, which is responsible of its microtubule nucleation capacity and is conserved in all members of the family, is actually formed by tandem repeats, covering well above 2/3 of the protein. We propose that this region forms a flexible solenoid involved in protein-protein interactions. Structural prediction and molecular modeling, combined with Circular Dichroism (CD) measurements reveal a predominant alpha-helical content. Furthermore, we identify full length homologs in fungi and shorter homologs with a different domain organization in diptera (including a paralogous expansion in *Drosophila*).

**Conclusions:**

Our results, represent the first computational and biophysical analysis of the TPX2 proteins family and help understand the structure and evolution of this conserved protein family to direct future structural studies.

**Electronic supplementary material:**

The online version of this article (doi:10.1186/s12900-016-0070-8) contains supplementary material, which is available to authorized users.

## Background

Spindle assembly involves the activities of multiple proteins that participate in localized microtubule nucleation, dynamics, and organization [1]. One of these proteins is TPX2 [2]. TPX2 was initially identified as a Microtubule-Associated Protein (MAP) responsible for the localization of the kinesin-like protein Xklp2 to microtubule minus ends during mitosis [3, 4]. In tissue culture cells, TPX2 is cell cycle regulated [5]. It accumulates in the nucleus during S/G2, at the spindle poles during mitosis, and it is degraded in early G1. In addition, experiments in *Xenopus* egg extracts have shown that TPX2 is regulated by the GTP-bound form of Ran [6] and triggers the local nucleation of microtubules around chromosomes. This activity is essential for spindle assembly in the presence or absence of centrosomes, in egg extract, and in tissue culture cells [5, 6]. Recently, it was found that TPX2 reduces microtubule growth and shortening by reducing the tubulin subunit off-rate from the microtubule tip [7].

TPX2 has other important functions, including a role in spindle pole organization [8] and in targeting and activating the mitotic kinase Aurora A [9–12]. Recent research points to other functions of TPX2 in non-mitotic cells [13]. For example, during interphase, TPX2 is recruited into the nucleus where it seems to have an alternative function in the cellular response to DNA damage [14]; a function in neurogenesis has also been shown [15]. Knowing the structure of TPX2 would help to understand the complex interactions and different locations of TPX2. However, little is known about the structure of TPX2 proteins.

Homologs of TPX2 were described in Chordata and in plants, where the function of the orthologue was functionally demonstrated [16]. But, the high sequence divergence of the TPX2 family of proteins and its presumed absence in other organisms has made difficult its phylogeny analysis to trace its emergence and evolution. The N-terminal Aurora A binding motif was identified in various species at the base of Metazoa (Placozoa: *Trichoplax adhaerens*) or even of Eukarya (Choanoflagellida: *Monosiga brevicollis*) [17].

Later, a homolog in drosophila, D-TPX2 (Ssp1/Mei-38), was identified that has low sequence similarity to the spindle-microtubule associated part but not to the Aurora A binding domain [18]. D-TPX2 localized with kinetochore microtubules in early mitosis and thus was proposed as the ortholog of TPX2. However, this proposed ortholog did not recapitulate most of TPX2 function. In addition, this short version of TPX2 was not found in ants, bee or wasp, which have instead the vertebrate/plant like version.

To complete the evolutionary and structural information on the TPX2 family we therefore decided to search for further homologues of TPX2, using sequence similarity analysis on sequence databases. We found further paralogs in drosophila and remote full length homologs in fungi. Additionally, our analysis revealed the presence of a variable number of tandem repeats in the C-terminal of all TPX2 related proteins making up a domain that covers more than 2/3 of the Chordata TPX2, which we predict to adopt an alpha-solenoid conformation. These findings have important consequences for our understanding of the interactions, functions and regulation of TPX2.

## Methods

### Computational sequence analysis

Initial identification of the repeats was done with HMMer [19] and we applied the REP algorithm [20] for the detection of all the instances of the repeat. Alignments were produced with HMMer [19], T-Coffee [21] and MUSCLE [22] using default parameters and were slightly refined manually. Phylogenetic trees and the image of the alignment were produced with ClustalW [23].

Protein secondary structure was predicted using the manually curated alignment of the repeats with Jnet (without homology search) [24] for different repeats of the human protein, and using full length human TPX2 with SABLE [25].

### Protein expression and purification

Full length *Arabidopsis* and *Xenopus* TPX2 were expressed as recombinant six-histidine tagged N-Terminal fusion proteins. Briefly, bacteria BL21(DE3) (Stratagene) cells were grown at an optical density of 0.7 (OD_600_) and induced for 5 h with IPTG at 1 mM. Bacteria were harvested by centrifugation and cell pellet were resuspended in a solution containing 15 mM imidazole, 20 mM HEPES, 150 mM KCl, 1 mM dithiothreitol (DTT), pH7.7 and 1 % Triton X-100. Cells were sonicated, centrifuged and the soluble fraction was incubated with 5 ml complete His-Tag Purification Resin (Sigma) at four degrees for 2 h with continuous inversion mixing. After 3 washes of lysis buffer, proteins were eluted with the same buffer containing 300 mM imidazole. Finally, proteins were further purified by size-exclusion chromatography with a Superdex 200 (GE Healthcare) equilibrated with 10 mM NaPO4, pH 7.4 and proteins concentration was determined by Bradford.

### CD spectropolarimetry

Circular dichroism (CD) spectra from TPX2 proteins (10 μM in 10 mM NaPO4, pH 7.4) were recorded on a on a Jasco-710 spectropolarimeter at 25 °C, over the wavelength range of 190 to 260 nm with 0.2 mm path. The spectra in the far-ultraviolet region required an average of ten scans and were subtracted from blank spectra performed with buffer [26, 27]. Secondary structure content was estimated using the K2D3 method [28]. The predicted percentages of secondary structure for atTPX2 and xlTPX2 indicated high alpha helical content (62 and 51 %, respectively) with some beta-sheet (15 and 14 % of beta-sheet, respectively).

### Three-Dimensional (3D) model prediction and validation

Structural modeling and visualization of the protein structure of TPX2 repeats were performed using iterative threading assembly refinement (I-TASSER software) [29]. Amino acid sequence (191–715) of each repeat from *X. laevis* TPX2 (accession number: AAF81694) was uploaded in FASTA format to I-TASSER and tertiary structures were predicted in PDB format for individual repeats. Energetic stability of each repeat was evaluated with FRST Energy Validation software [30] and each repeat model was examined for its compatibility with the sequence alignment. An initial structural model of *X. laevis* TPX2 was assembled with PyMol (version v1.7.2 software) [31]. Then the model was further refined by an iterative procedure. To validate the structural model, we assessed its quality in terms of covalent bonds, packing, torsion angles and flexibility.

## Results

### Identification of TPX2 homologs

**Fig. 1:**
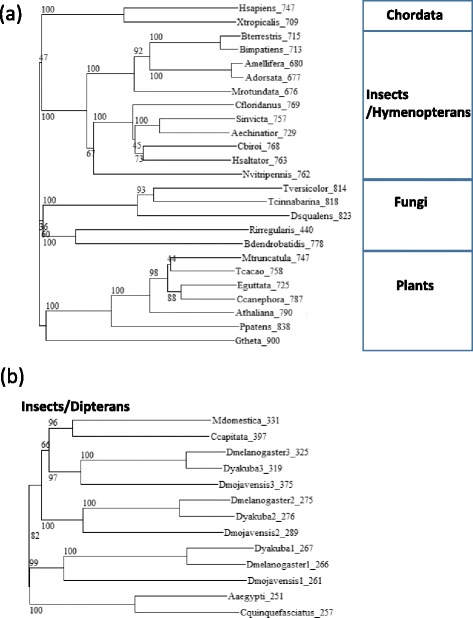
Phylogenetic trees of TPX2 homologs. **a** Phylogenetic tree of full length orthologs of TPX2 in representative species. **b** Phylogenetic tree of short orthologs of TPX2 in representative dipteran species. *Drosophila* has three paralogs. The labels indicate species and length of the protein. Numbers in the tree represent bootstrapping values. The sequences and NCBI identifiers are available as Additional file 1 and Additional file 3 for (**a**) and (**b**), respectively. The multiple sequence alignments used to do the phylogenetic trees are available as Additional file 2 and Additional file 4 for (**a**) and (**b**), respectively

We searched the sequence databases for putative homologs of vertebrate TPX2. In addition to the previously described homologs, we found full length homologs in multiple fungal species without a clear pattern in their taxonomic distribution. Differently, we appreciated a clear taxonomic pattern within insects, where whole length orthologs were found in hymenopterans (Fig. 1a), while dipterans (including the fly) had a shorter version (Fig. 1b). In addition to D-TPX1 we could identify two other paralogs in *Drosophila* (Fig. 1b).

Multiple sequence alignment of the TPX2 protein homologs revealed a highly conserved C-terminal region. The N-terminal Aurora A binding motifs identified in [16] aligned also with the fungal and hymenopteran homologs (Additional file 2).

For comparison, there are currently three entries in the PFAM database of protein domains (as of 20 April 2016; [32]) covering human TPX2. Aurora-A bind (PF09041), matching aa 1–68, is found in 43 species restricted to Euteleostomi (e.g., fish, coelacanth and Tetrapoda), and thus seems not to cover all the sequences having the Aurora A binding motif. The other two have much wider distributions. TPX2_importin (PF12214) matching aa 361–489 and TPX2 (PF06886) matching 662–718, are found in 113 and 145 species, respectively: in plants, Stramenopiles (algae), fungi (3 and 22 species, respectively, not in e.g., *Saccharomyces cerevisiae*), Alveolata (*Tetrahymena thermophila*), Choanoflagellida (*Monosiga brevicollis*) and diverse Metazoa.

The PFAM hits do not match dipteran homologs, reflecting their divergence from the long version of TPX2. Neither PFAM hits, nor our own results included matches in *Caenorhabditis* species.

### The C-terminal part of the TPX2 homologs shares a series of repeats

**Fig. 2:**
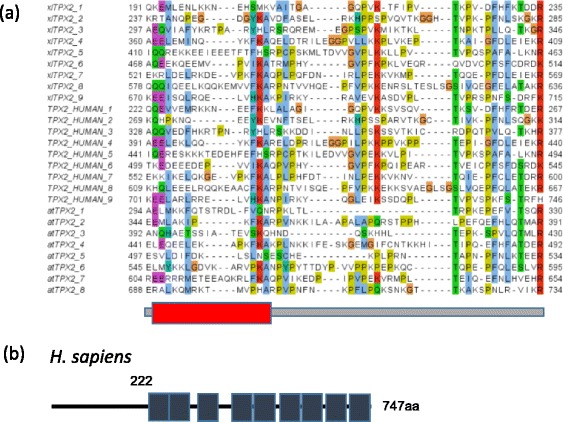
Repeats in TPX2 proteins. **a** Multiple sequence alignment of tandem repeats in *Xenopus laevis*, human and *A. thaliana* TPX2. The red box indicates a summary of predictions for an alpha-helix (see Methods for details). **b** Position of repeats in human TPX2. UniProt database identifiers are Q6NUF4 for xlTPX2, Q9ULW0 for TPX2_HUMAN, and F4I2H7 for atTPX2. The multiple sequence alignment is available as Additional file 5

Careful inspection of the alignment of the TPX2 homologs indicated a number of blocks of conservation that were apparently repeated in several parts of the alignment. As this was an indication of putative protein repeats, we followed an iterative procedure to define these repeats, align them, and identify new ones within these sequences. The multiple sequence alignment of TPX2 repeats from human, *Xenopus laevis* and *Arabidopsis thaliana* is displayed in Fig. 2a.

The repeat length is of about 50 aa, and the occurrence of nine repeats in tandem at the C-terminal of these sequences, plus small inserts between repeat units, results in a domain of about 500 aa, thus covering the majority of full length TPX2 (Fig. 2b).

Computational prediction of secondary structure of the repeat region indicated the presence of alpha-helical structure in the first half of the repeat (red box in Fig. 2a). No coherent predictions could be obtained for the rest or the repeat unit.

### The C-terminal part of the TPX2 is α-Helical

**Fig. 3:**
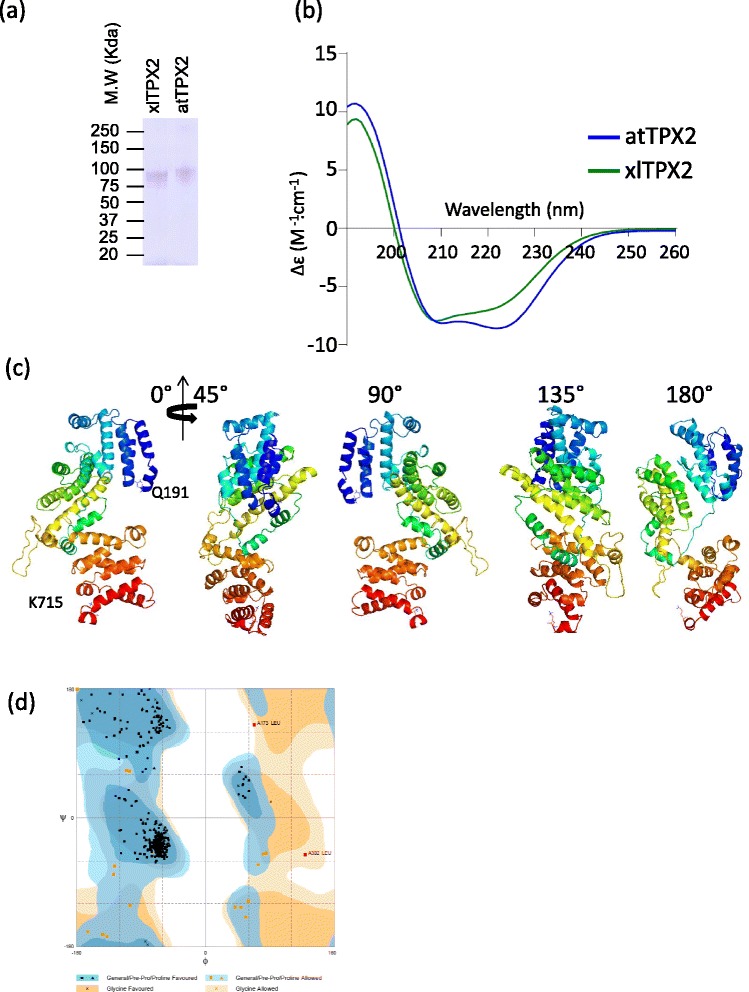
Biochemical and structural analysis of TPX2. **a** SDS-PAGE analysis of *Xenopus* and *Arabidopsis* TPX2 proteins. **b** Spectra in the region of 260–190 nm were obtained at 25 °C for full length xlTPX2 and atTPX2. Both spectra present a typical alpha helical profile with two minima (λ208 and λ222 nm). **c** Molecular model of xlTPX2 (Q191-K715) represent a compact structure of repeated α-helices linked by a flexible loop. **d** Ramachandran plot of the xlTPX2 model. About 96 % of all residues were in favored regions, and about 4 % of the residues were in an allowed region. Two outliers were found, Leucines at positions 173 and 302, although, visual inspection did not reveal any steric clash

To validate our computational predictions, we generated recombinant *Xenopus laevis* and *Arabidopsis thaliana* TPX2 proteins (xlTPX2 and atTPX2, respectively). After purification, proteins were submitted to SDS-PAGE followed by coomassie staining to assess their degree of purity (Fig. 3a). Both proteins migrated as a single band at around 90 KDa, as expected by the predicted molecular weight for xlTPX2 (82383 Da) and atTPX2 (86477 Da).

To investigate TPX2 secondary structure, both proteins were analyzed by circular dichroism (CD). Interestingly, CD spectra in the far-UV region, revealed two ellipticity minima at 208 nm and 222 nm, characteristic of proteins with high alpha-helix structure content (Fig. 3b). The predicted percentages of secondary structure for atTPX2 and xlTPX2 indicated an alpha helical content of 69 and 68 %, respectively, connected by coil or unstructured region structure accounting for 31 and 34 % of the secondary structure. These findings are consistent with the alignment generated and the presence of a conserved repeat in the C-terminal of TPX2. To further validate our findings we performed three dimensional structural modeling of xlTPX2 C-terminal domain (Fig. 3c). The TPX2 model, was generated by modelling each repeat defined in Fig. 2a. For each individual repeat, amino acid sequences were uploaded in FASTA format to I-TASSER. The predicted structures for each repeat were selected based on the confidence score (c-score), energetic stability and its compatibility with the structural prediction obtained by sequence alignment. Consequently, the final model was built with homology to the following structural PDB templates: 3x29A, 4cgyA, 4h0sA, 2be4A, 4ixjA, 1ad6, 2q1fA, x4by6A and 5J0H. The average values of the models for all the repeats were c-score = −0.3, estimated TM-score = 0.53 ± 0.15 and estimated RMSD = 5.5 ± 3.5 Å. The structural model of xlTPX2 was further refined, using an iterative procedure with 3Drefine [33] and GalaxyRefine software [34]. The quality and validity of the structural model was confirmed using the Protein Structure Evaluation Suite & Server [35]. Upon analysis, we could confirm that the structural model was coherent based on its Ramachandran diagram (Fig. 3d), obtained with the Rampage software [36]. Interestingly, no beta strands were visualized in these repeats, and predominance of alpha helical content was calculated as seen by the three dimensional representation and Ramachandran plot (Fig. 3c and d). Taken together, these results suggest that TPX2 repeats are mostly composed of alpha helices, although experimental structural validation will be needed to confirm this result.

## Discussion

The TPX2 family of proteins of microtubule nucleators seems to be present in full length (with an Aurora A binding site) in most eukaryotic species, with apparent loses in scattered taxa (e.g., *S. cerevisiae* and *C. elegans*) or substitutions for shorter forms (e.g., dipterans). TPX2 proteins share a C-terminal region, which is necessary and sufficient for their activity in triggering microtubule nucleation [37]. Our computational and biophysical analysis of TPX2 shows that this region is composed of a variable number of tandem repeats containing alpha-helical segments. There are many structural repeats formed by alpha-helices (e.g., Armadillo, HEAT, TPRs) that are often involved in protein-protein interactions [38] and have been described as alpha-solenoids [39]. At a length of 50 amino acids, TPX2 repeats fit very well with the lengths described for these types of repeats. We hypothesize that TPX2 interacts in this region with one or more protein partners, being this interaction crucial for TPX2-mediated microtubule nucleation.

A 3D structure of a complex between a *Xenopus laevis* TPX2 fragment in the repeat region was solved in complex with importin-alpha (PDB:3knd) [40]. This shows that the nuclear localization signal in *X. laevis* TPX2 284-KRKH-287 is recognized by importin-alpha (bound to the minor NLS-binding site). The fragment used was 270–350 because smaller fragments did not give good quality crystals for X-ray crystallography. Residues 327-KMIK-330 were bound to another recognition site in importin-alpha (major NLS-binding site). We take the fact that the fragment that successfully formed a crystal includes a full repeat (see Fig. 2a) as an indication that our definition of the repeat unit is correct. In this structure, the TPX2 fragment is solved in only two stretches that are extended and seem not to adopt secondary structure. The arrangement of the fragments does not agree with an expected folded structure of the repeat where the start and end occupy positions near in space, as it is usual in structural units of tandem repeats [38]. We hypothesize that the nuclear localization signal in TPX2 is hidden and becomes exposed upon interaction with other molecules which would disorganize the repeat unit holding it (repeat #3 in *Xenopus*) separating the repeats 1–2 from the 4–9. We have proposed a similar unfolding mechanism of tandem repeats triggered by phosphorylation for the mineralocortocoid receptor, which contains a region with tandem repeats that holds multiple phosphorylation sites [41]. Similarly, human TPX2 has a number of phosphorylation sites in the tandem repeat region that are cell cycle dependent (serines 292, 293, 486 and 738; [42]). This could be a general mechanism by which the structural flexibility of tandem repeats could be exploited.

Here, by finding full length homologs in fungi and other primitive unicellular species, we have completed the phylogenetic distribution of the TPX2 family, which seems to have appeared very early after the emergence of eukaryotic organisms, pointing to an ancient and crucial function in the organization of cell division. Regardless, it has been noted that the variability of domain organization of the members in this family suggests that while TPX2 functions might be widely conserved in Eukarya, diverse functional modules could be placed in different proteins or functions performed by other protein families [13]. TPX2 apparent absence in many fungal species, or its replacement with a shorter version in dipterans agrees with this.

Accordingly, although a homolog of TPX2, TPXL-1, was identified by homology to the Aurora A kinase binding domain in *C. elegans* [43], the authors were not able to demonstrate the nucleation activity of this protein characteristic of the TPX2 proteins family [2] and the sequence similarity to the Aurora A kinase binding site was challenged [17]. Therefore, there is not enough evidence to claim that this particular *C. elegans* protein is a TPX2 homologue [44] and thus *Caenorhabditis*, like dipterans, seems to have got away with a different system to substitute TPX2 function.

In all, our analyses suggest a structure for a large fraction of the TPX2 protein while stressing their evolutionary flexibility. The tandem repeat region could be involved in transient protein-protein interactions regulated by cell-cycle dependent phosphorylation. We trust that this information will be helpful to direct future experiments in any of the members of this taxonomically widely distributed family.

## Conclusions

Here, we have characterized a novel repeat region in the spindle pole protein TPX2. We predict that this region folds into a domain composed of an ensemble of alpha-helical tandem repeats. This region covers more than 2/3 of the protein, thus this is an important result since so far there is absolutely no structural information regarding TPX2.

## Additional files

Additional file 1: FASTA file of sequences used for Fig. 1a. (TXT 21 kb)
Additional file 2: FASTA file of sequences used for Fig. 1a. (TXT 5 kb)
Additional file 3: Multiple sequence alignment used in Fig. 1a. (TXT 43 kb)
Additional file 4: Multiple sequence alignment used in Fig. 1b. (TXT 10 kb)
Additional file 5: Multiple sequence alignment used in Fig. 2. (TXT 2 kb)

## Abbreviations

CD: Circular dichroism
MAP: Microtubule-associated protein
TPX2: Targeting protein for Xklp2
